# Effect of Cobalt, Nickel, and Selenium/Tungsten Deficiency on Mesophilic Anaerobic Digestion of Chemically Defined Soluble Organic Compounds

**DOI:** 10.3390/microorganisms8040598

**Published:** 2020-04-20

**Authors:** Luka Šafarič, Sepehr Shakeri Yekta, Bo H. Svensson, Anna Schnürer, David Bastviken, Annika Björn

**Affiliations:** 1 Department of Thematic Studies–Environmental Change, Linköping University, SE-581 83 Linköping, Sweden; sepehr.shakeri.yekta@liu.se (S.S.Y.); bo.svensson@liu.se (B.H.S.); anna.schnurer@slu.se (A.S.); david.bastviken@liu.se (D.B.); annika.bjorn@liu.se (A.B.); 2 Biogas Research Center, Linköping University, SE-581 83 Linköping, Sweden; 3 Department of Molecular Science, Swedish University of Agricultural Science, Uppsala BioCenter, SE-750 07 Uppsala, Sweden

**Keywords:** artificial substrate, biogas, trace elements, micronutrients, volatile fatty acids, kinetics

## Abstract

Trace elements (TEs) are vital for anaerobic digestion (AD), due to their role as cofactors in many key enzymes. The aim of this study was to evaluate the effects of specific TE deficiencies on mixed microbial communities during AD of soluble polymer-free substrates, thus focusing on AD after hydrolysis. Three mesophilic (37 °C) continuous stirred-tank biogas reactors were depleted either of Co, Ni, or a combination of Se and W, respectively, by discontinuing their supplementation. Ni and Se/W depletion led to changes in methane kinetics, linked to progressive volatile fatty acid (VFA) accumulation, eventually resulting in process failure. No significant changes occurred in the Co-depleted reactor, indicating that the amount of Co present in the substrate in absence of supplementation was sufficient to maintain process stability. Archaeal communities remained fairly stable independent of TE concentrations, while bacterial communities gradually changed with VFA accumulation in Ni- and Se-/W-depleted reactors. Despite this, the communities remained relatively similar between these two reactors, suggesting that the major shifts in composition likely occurred due to the accumulating VFAs. Overall, the results indicate that Ni and Se/W depletion primarily lead to slower metabolic activities of methanogenic archaea and their syntrophic partners, which then has a ripple effect throughout the microbial community due to a gradual accumulation of intermediate fermentation products.

## 1. Introduction

Anaerobic digestion (AD) is an important biotechnology for combining organic waste treatment with renewable energy recovery [1]. In AD processes, polymers in organic waste are degraded by facultative and strict anaerobic bacteria to oligomers and monomers, which are subsequently fermented, e.g., to volatile fatty acids (VFAs), alcohols, CO_2_, and H_2_. Fermentation products are further oxidised to acetate, CO_2_ and H_2_, or formate, which are the main sources for methanogenesis [2,3]. Availability of specific trace elements (TEs), which serve as micronutrients for the growth and activity of microorganisms at different stages of AD, is central for the efficiency and stability of AD processes [4,5,6]. Cobalt (Co) is essential for the functioning of acetogens and methanogens due to its involvement in cobamide coenzymes, responsible for methyl group transfers [7,8]. Nickel (Ni) is vital for methanogenesis due to its functional role in porphyrinoid F_430_, a prosthetic group of the active site of methyl coenzyme M reductase, which catalyses the formation of methane [9]. Nickel is also involved in other enzymes, such as Ni–Fe hydrogenases and the carbon monoxide dehydrogenase/acetyl-CoA synthase in acetoclastic methanogenesis, as well as energy-converting hydrogenases and F_420_-reducing hydrogenases during hydrogenotrophic methanogenesis and acetogenesis [10]. Selenium and tungsten (Se and W) are particularly important for acetogens and hydrogenotrophic methanogens due to their role in the function of hydrogenases and formate dehydrogenase, which transforms formate to CO_2_ and H_2_ [10,11]. 

A deficiency of TEs commonly results in alterations of microbial community composition and function, suboptimal AD operation, and eventually complete process failure [12,13,14,15,16,17]. Accordingly, supplementation of TEs is needed in many cases to maintain the activity of the microbial community in AD systems. Deficiencies of TEs may occur either due to insufficient TE content in the substrate or a high concentration of compounds which bind the TEs, decreasing their bioavailability [5]. Examples of substrates commonly lacking specific TEs is the organic fraction of municipal solid waste, or energy crops, whose digestion can often be improved by addition of Fe, Ni, Co, and Se [18,19,20,21]. Sulphur-rich substrates, such as grain stillage, on the other hand, often result in decreased bioavailability of the present TEs [6,22]. In cases like this, additional supplementation of Fe may be needed in order to bind the sulphur and increase the bioavailability of other TEs [23]. As a result of substrate diversity, the TE requirements reported in the literature typically vary. Co is reported to be required in ranges of 0.7–14 µg per g of chemical oxygen demand fed to the system (COD_fed_) and 3.6–25 µg/g COD_fed_ in acidogenic and acetogenic/methanogenic pathways, respectively. Nickel requirements range from 2.3 to 4.8 µg/g COD_fed_ for the acidogenic pathways and from 0.9 to 5.4 µg/g COD_fed_ for the acetogenic and methanogenic pathways [17,18,19,24]. Furthermore, stable acidogenesis requires Se and W at 48 µg/g COD_fed_ and 1.65 µg/g COD_fed_, respectively, while acetogenic/methanogenic microorganisms seem to require 9.6 µg/g COD_fed_ for Se and 0.02–0.33 µg/g COD_fed_ for W [17,18,19,24].

The aforementioned levels of TEs are based on a range of different methanogenic systems with varying substrate complexity from AD of organic wastes to pure culture enrichments on synthetic growth media, which likely explains the relatively broad ranges of TE requirements. The inherent variability in TE requirements of different AD systems primarily originates from utilization of complex substrates that affect the TEs via interactions between the substrate matrix and the TE, modulating its bioavailability and microbial uptake [5]. The use of pure cultures, on the other hand, allows for conducting controlled experiments with respect to TE requirement by the microorganisms, yet limiting the extrapolation of the results to AD of complex organic matter. Benefits from both approaches can be attained by starting with a mixed microbial community performing AD of a complex substrate and substituting the substrate with a defined soluble one, thus washing out the complex substrate matrix over time. This eliminates the effects of substrate variability, while still preserving all the typical metabolic pathways that are universally present in AD processes, regardless of substrate complexity [25].

Thus, this study aims to elucidate the effect of TE deficiency (in particular, Co, Ni, and the combination of Se and W) on a mixed anaerobic microbial community enriched on chemically defined soluble compounds. This provides important information on the TE requirements of complex microbial communities with minimal effects from the chemical complexation with the substrate matrix. As a result, this knowledge can be applied together with information on substrate composition to more accurately determine the optimal supplementation levels for a range of AD systems, as well as to provide information needed to improve mathematical models of AD [26]. Finally, shifts in the microbial community composition were monitored in this study together with VFA- and methane production dynamics to elucidate the effects of TE deficiency on the system and provide further insights on the dynamics of TE-induced process disturbances.

## 2. Materials and Methods

### 2.1. Experimental Setup

Four mesophilic (37 °C) continuous stirred-tank biogas reactors (CSTBRs; R_ctrl_, R_Co_, R_Ni_, and R_SeW_) with a working volume of 4 L were started by inoculation from a 9 L reactor, which had operated at steady state conditions for three years, as described by Speda et al. [25]. The reactors were fed with a chemically defined substrate, containing essential mineral nutrients and simple soluble energy and carbon sources (glucose, sucrose, lysed casein, VFA, and alcohols) identical to the one of the original reactor. Detailed information about substrate composition and the concentration of individual components is presented in the Supplementary Information (Table S1). Operational conditions were similar to those of the original reactor, with a hydraulic retention time (HRT) of 30 days and an organic loading rate (OLR) of 1.7 gCODL^−1^d^−1^. Trace elements, including Co, Ni, Se, and W were supplemented at initial concentrations of 0.32, 0.13, 0.07, and 0.08 nM, respectively, which corresponds to 0.36, 0.15, 0.11, and 0.28 ng/g COD_fed_. The specific supplementation levels were chosen to be identical to those of the reactor used for inoculation. An accidental high dose of Se and W was fed to all reactors on days 109 and 110 without affecting process performance. After a stabilisation period of 206 days, i.e., more than 6 HRTs, Co and Ni supplementation was eliminated from the substrates of R_Co_ and R_Ni_, respectively, while both Se and W were eliminated in the case of R_SeW_ due to their combined importance for the activity of specific enzymes, such as formate dehydrogenase [11]. The control reactor, R_ctrl_, received the same substrate as before. As a result, TEs were gradually washed-out from reactors R_Co_, R_Ni_, and R_SeW_ with an expected reduction of more than 95% of their initial supplemented concentrations after 3 HRTs. The reactors were operated for a total of 350 days, with the TE depletion lasting for 144 days.

### 2.2. Process Monitoring

Biogas production was measured continuously with gas meters working on the principle of fluid displacement (Ritter GmbH, Bochum, Germany). The volume of gas produced was re-calculated to dry normal conditions (0 °C, 1 atm.) with the “biogas” package in R (R foundation for statistical computing, Vienna, Austria) [27]. Methane concentrations were monitored continuously with methane sensors based on absorption of infrared light (BlueSens gas sensor GmbH, Herten, Germany). The data from the gas sensors and the gas meters were combined to calculate the daily methane production dynamics. Piecewise regression was used to model the abrupt changes in gas production kinetics, identified as “breakpoints”; i.e., the points where lines of two fitted linear models would intersect [28].

Total solid (TS) and total volatile solid (TVS) contents were measured weekly by drying (105 °C) and burning (550 °C) in accordance with the Swedish Standard Method (SS-028113). VFA concentrations, which served as proxies for evaluating the degradation of fermentation products, were monitored weekly by gas chromatography with a flame-ionisation detector (GC-FID; Hewlett Packard, San Jose, CA, USA), according to Jonsson & Borén [29], and pH was measured with an InoLab pH meter (WTW, Weilheim, Germany) according to the Swedish Standard Method (SS-12176). On three occasions, selected based on reactor performance (days 153, 237, 340), eight sludge samples were extracted from each reactor over a 24-h period and analysed for VFAs in order to monitor intra-daily VFA kinetics upon feeding and compare them with changes in methane production rates. 

The concentrations of TEs (Co, Ni, Se, and W) in the main substrate components (glucose, sucrose, casein, tap water, vitamin, and trace element solutions) were measured by inductively coupled plasma mass spectrometry (ICP-MS, NexION 300, Perkin Elmer, Waltham, MA, USA). The concentrations of the individual TE in the reactors were calculated based on the levels of their supplementation, as well as the contents in each of the main substrate components (Table 1). This was done to provide more accurate TE concentration estimates for each reactor, since direct measurements could not be accurately performed due to the low concentrations induced by TE depletion. Since the exact speciation and resulting bioavailability of TE from different sources is unclear, and since the contributions of substrate components without TE solutions were constant throughout the experiment, the calculated effects of depletion on TE concentrations are reported in the results as the predicted concentration of the supplemented fraction, which was in ionic form (i.e., TE and Se/W solutions).

Summaries of the process parameters were calculated for each reactor by defining separate periods based on reactor performance and calculating the means and standard deviations from all data available within each period. Statistical comparisons between the different reactors were performed either by Student’s *t*-test or the Tukey honest significant difference test (Tukey HSD), depending on the number of values being compared.

### 2.3. Microbial Community Analyses

Samples from periods with major changes in reactor performance were selected for DNA extraction, i.e., days 104, 118, 201, 222, 257, 285, 320, and 348, corresponding to samples collected before and after the accidental addition of Se/W, before the start of TE depletion, before propionate accumulation, before acetate accumulation, after TVS increase, before butyrate accumulation, and after process failure in R_Ni_ and R_SeW_. Sequencing of 16S rRNA gene amplicons was performed with two primer pair sets; 515′F/805R for amplification of prokaryote 16S rRNA genes [30], and 516F/915R for amplification of archaeal 16S rRNA genes [31]. The raw sequences were deposited in the Sequence Read Archive of the National Centre for Biotechnology Information (NCBI) under the bio-project identification number: PRJNA515619.

Bacterial amplicon sequence variants (ASVs) obtained with the first primer set and archaeal ASV obtained with the second primer set were selected and analysed separately. Specific details on the DNA extraction, sequencing, and data treatment have been described previously [32] and are presented in the Supplementary Information (Text S1). The bacterial and archaeal communities were analysed for community diversity and evenness (*^1^D* and *^1^E*) by calculating the first-order Hill diversity numbers, which have been suggested to be the most important diversity and evenness indices [33]. Non-metric multidimensional scaling (NMDS) based on the “Bray–Curtis” dissimilarities was used to evaluate at which point the microbial communities of TE-depleted reactors began diverging from the control. Deeper insights into the archaeal and bacterial community compositions were retrieved by evaluating the relative abundances of different microbial groups at different taxonomic levels. Clustering analysis was used to evaluate which samples were similar in their bacterial and archaeal community compositions. This was performed by first standardising the data by the “Hellinger” method [34] and then clustering with the “K-means” approach using the “vegan” package [35] in R [36]. The decision on the optimal number of clusters was based on the combination of the evaluations of dendrograms produced by “Bray–Curtis” distance hierarchical cluster analysis and the “Calinski–Harabasz” approach [37]. Analysis of variance (ANOVA) was used to determine whether the obtained clusters differed significantly in relation to their associated process parameters. Indicator species analysis [38] was performed on the genus level in order to identify genera significantly correlated with each cluster. 

## 3. Results

### 3.1. Effects of TE deficiency on Conversion of Soluble Substrate to Biogas

**Figure 1:**
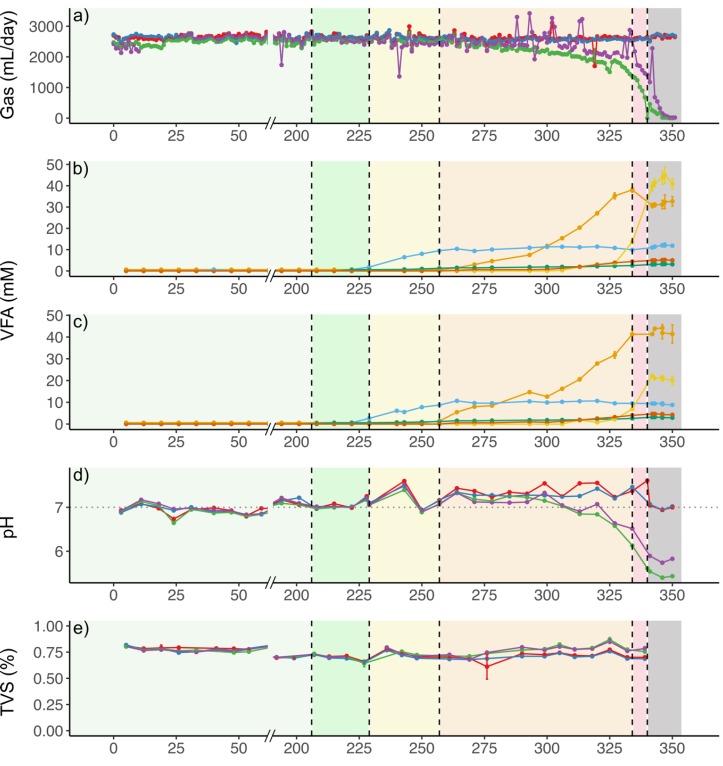
Overview of the experiment performance: (**a**) standardised daily biogas production of all reactors (mL/day); (**b**) volatile fatty acid (VFA) concentrations in R_Ni_* (mM); (**c**) VFA concentrations in R_SeW_* (mM); (**d**) pH of all reactors; (**e**) total volatile solids of all reactors (% of wet mass). Background colours signify the different periods in the experiment: light green, stabilisation period before the experiment; dark green, beginning of TE depletion in R_Co_, R_Ni_, and R_SeW_; yellow, increase of propionate concentrations in R_Ni_/R_SeW_; orange, increase of acetate concentrations in R_Ni_/R_SeW_; red, increase in butyrate concentrations in R_Ni_/R_SeW_; grey, process failure of R_Ni_/R_SeW_. The days between 50 and 200 are not shown, since all parameters remained relatively constant. Legend for (**a**,**d**), and (**e**), colours denote reactors: red, R_ctrl_; blue, R_Co_; green, R_Ni_; purple, R_SeW_. Legend for (**b**,**c**) colours denote VFA: orange, acetate; blue, propionate; red, iso-butyrate; yellow, butyrate; green, iso-valerate. * VFA concentrations in R_ctrl_ and R_Co_ were ≤0.6 mM throughout the experiment and are therefore not presented here.

Before starting the experiment (days 0–206), biogas production was similar among the reactors with an average value of 2600 ± 110 mL per day (Figure 1a), at a methane concentration of 55% ± 3%, corresponding to a methane production of 190 ± 14 mL gCOD_in_^−1^. The theoretical methane potential of the synthetic substrate, calculated according to the Buswell formula, is 350 mL gCOD_consumed_^−1^, implying that 56% ± 4% of theoretical methane potential was reached at steady state condition before the induction of TE deficiency. Operational performance of R_ctrl_ and R_Co_ remained at stable state in terms of biogas production, VFA concentrations, pH, and TVS contents throughout the experiment (Figure 1). Thus, omission of CoCl_2_ and Co-containing vitamin B_12_ from the synthetic substrate of R_Co_ did not lead to major changes in process performance. In contrast, operational performance of R_Ni_ and R_SeW_ deteriorated soon after supplementation of Ni and Se/W was omitted from the corresponding substrates. The biogas production in R_Ni_ and R_SeW_ after removing the TE was 2500 ± 75 and 2500 ± 79 mL day^−1^, respectively, which was slightly, but not significantly lower than that of the control (2600 ± 74 mL day^−1^ between days 224 and 230; *p* = 0.09, Tukey HSD). Accordingly, lower methane yields of 53% ± 3% and 53% ± 2% of the theoretical potential were obtained in R_Ni_ and R_SeW_ compared to that of R_ctrl_ during this period (60% ± 2% of theoretical potential). Methane concentrations began to slightly decrease from 53% ± 3% to 51% ± 3% and from 53% ± 4% to 51% ± 3% in R_Ni_ and R_SeW_, respectively, while the concentrations increased in R_ctrl_ from 56% ± 3% to 57% ± 3%, and remained fairly stable in R_Co_ at 55% ± 3%.

Furthermore, propionic acid began to accumulate from day 229, when concentrations of Ni and Se/W fractions, originating from supplementations, were 0.06 and 0.12/0.13 nM in R_Ni_ in R_SeW_ respectively (i.e., 2 ng Ni gCOD_fed_^−1^, and 6 ng Se gCOD_fed_^−1^ and 14 ng W gCOD_fed_^−1^). After 28 days of accumulation, the propionate levels stabilised at 11 ± 0.9 and 10 ± 0.6 mM in R_Ni_ and R_SeW_ (corresponding to 820 ± 67 and 740 ± 44 mg/L), respectively, when supplemented Ni concentrations were 0.02 nM in R_Ni_ and supplemented Se/W concentrations were 0.05/0.05 nM in R_SeW_ by day 257. Concurrently with the stabilization of the propionate concentration, acetate started to accumulate (Figure 1b,c), reaching 38 ± 1.1 and 41 ± 2.4 mM in R_Ni_ and R_SeW_ (corresponding to 2300 ± 66 and 2500 ± 140 mg/L), respectively at day 334. Thereafter, butyrate concentration increased in both reactors until day 341, when the biogas production halted (Figure 1). This was also associated with a substantial decrease of methane concentrations from 47% ± 5% and 47% ± 4%, to 29% ± 3% and 35% ± 2% in R_Ni_ and R_SeW_, respectively. Towards the end of the experiment, butyric acid concentrations reached 40 ± 2.1 mM (equivalent to 3500 ± 190 mg/L) in R_Ni_ and 22 ± 0.11 mM (equivalent to 1900 ± 10 mg/L) in R_SeW_. 

### 3.2. Effects of TE Deficiency on Kinetics of VFA Turnover and Biogas Formation

**Figure 2:**
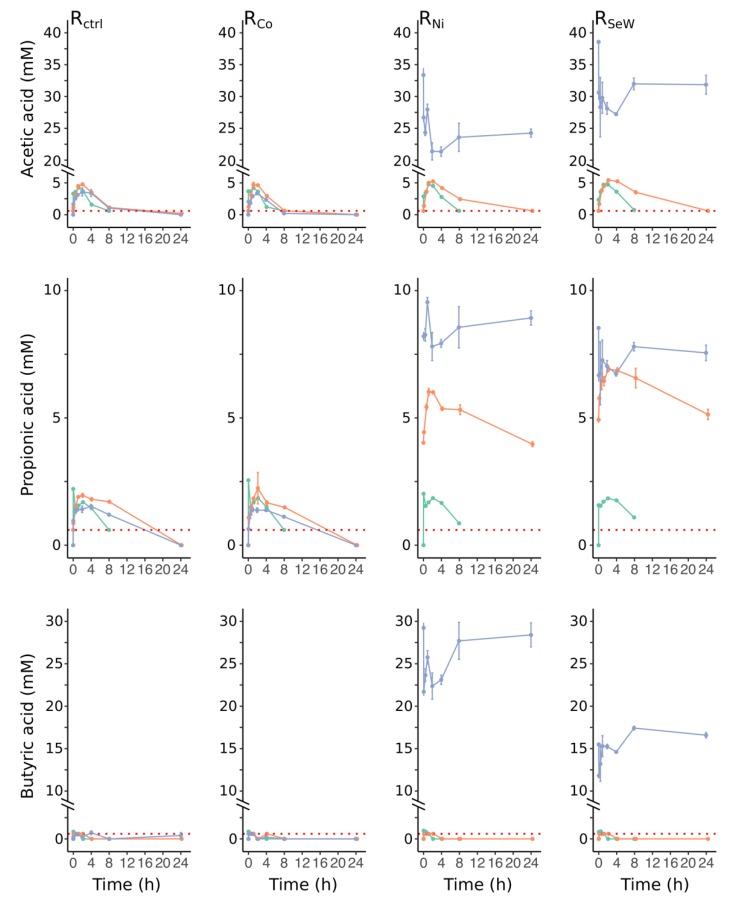
VFA dynamics over a 24-hour feeding cycle. The colours denote separate sampling occasions: green, day 153; orange, day 237; blue, day 340. The horizontal red dotted line denotes the method’s limit of quantification (i.e., the values on or below it are considered detected but not quantified). Measurements on day 153 (green lines) were performed only for the first 8 h after feeding.

The pH was stable at 7.1 ± 0.1 in reactors R_Ni_ and R_SeW_ until day 306, but gradually decreased in parallel with an increase in the acetate concentration, followed by a rapid drop to <5.7 between days 334 and 346 (Figure 1d). Analysis of the daily VFA turnover kinetics demonstrated that acetic, propionic, and butyric acid concentrations temporarily increased between feedings in the reactors and were depleted within 8 h after feeding during the stabilisation period (green lines in Figure 2). Upon TE depletion, the acetic acid concentrations in R_Ni_ and R_SeW_ had slightly higher daily maxima between feedings and longer turnover times as compared to the control reactor. However, acetate was completely consumed within the feeding cycle, indicating that the consumption rate of acetate exceeded its production rate. After the removal of Ni and Se/W supplementation from the substrate of R_Ni_ and R_SeW_, propionic acid was only partially consumed between two feeding occasions and began to gradually accumulate from one day to another (orange lines on Figure 2). A notable feature in the daily behaviour of acetic, propionic, and butyric acid on day 340 (Figure 2) was that their concentrations dropped directly after feeding, which can be attributed to dilution of the reactor medium due to sludge removal and substrate addition. Nevertheless, the daily production of butyrate and propionate from fresh substrate compensated for the dilution effect, resulting in a net increase of their concentrations.

**Figure 3:**
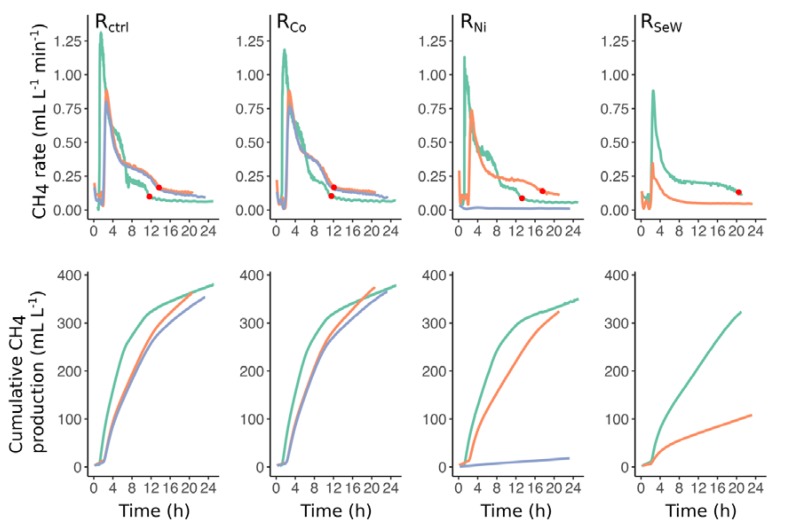
CH_4_ production rates and cumulative CH_4_ production on days 154 (green), 238 (orange), and 340 (blue). The red dots identify the breakpoints, monitored in this study, signifying the shift of methane rates to a residual level. Data for reactor R_SeW_ on day 154 were not available due to technical problems with the sensor. The lines represent the running mean across 15 min of reactor operation.

**Figure 4:**
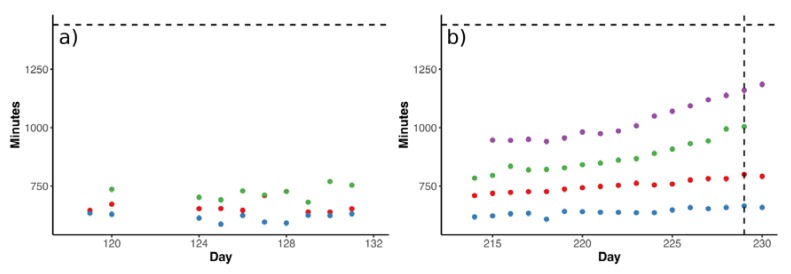
Breakpoint, signifying the time (in minutes) after substrate dosing when the majority of VFA was consumed. (**a**) Time required for the consumption of VFA before TE depletion; (**b**) time required for the consumption of VFAs after TE depletion (from day 206). The vertical dashed line corresponds to the day when propionate accumulation was detected in R_Ni_ and R_SeW_. The horizontal dashed line represents 24 h after substrate dosing. Colours denote reactors: red, R_ctrl_; blue, R_Co_; green, R_Ni_; purple, R_SeW_.

Throughout the day, methane production proceeded at distinctly different rates (Figure 3). We observed that there were several points during the day when the rate of methane production abruptly changed. In particular, the point when methane production rate reached a steady level corresponded to the time after feeding when all VFAs were mostly depleted in the reactors (red dots on Figure 3). The dynamics of this transition point can be monitored by extracting the “breakpoint” from piecewise regression applied to the data, representing the time when methane production is constant at its minimum level and the VFA concentration is equal to or close to zero. The gas production rates remained relatively stable before TE depletion, reaching the breakpoint around 12 h after substrate dosing (Figure 4a). 

Upon Ni and Se/W depletion in R_Ni_ and R_SeW_, the time of the breakpoint started to increase, beginning around one week before propionate accumulation (day 223; Figure 4b), indicating that it took longer to reach the steady methane production associated with VFA depletion. This observation is also in line with the slower kinetics of VFA turnover observed after inducing TE deficiency (Figure 2). Furthermore, the daily pattern of methane production rates gradually shifted from a stepwise cascade to an exponential decrease trend upon TE depletion in R_Ni_ and R_SeW_ as the breakpoints drifted out of the 24-h window. Therefore, most of the transitions between different gas rates throughout the day disappeared (Figure 3), leaving the initial peak of methane production as the most prominent feature of the daily patterns.

### 3.3. Effects of TE Deficiency on Bacterial Community

**Figure 5:**
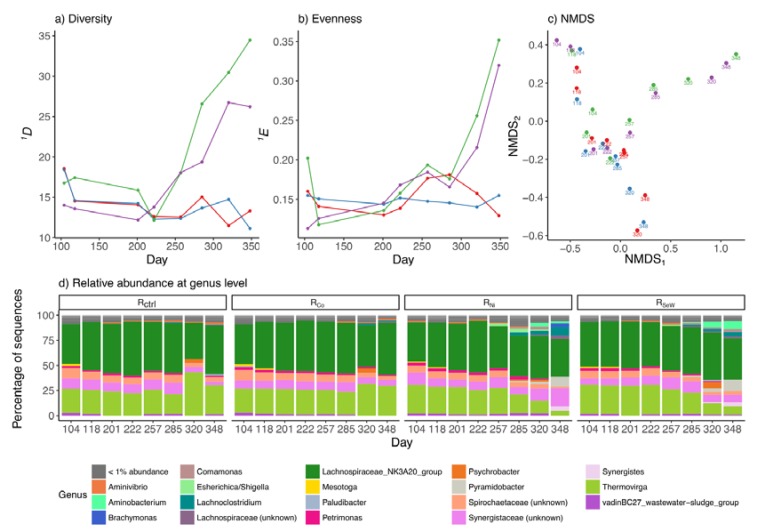
Results of the bacterial community analyses: (**a**) Hill diversity of order 1 (^1^D); (**b**) evenness of Hill order 1 (^1^E); (**c**) Non-metric multidimensional scaling (NMDS); (**d**) relative abundances for each reactor at the genus level. Sequences with a relative abundance of <1% are grouped together and marked as “<1% abundance”. Legend for (**a–c**), colours denote reactors: red, R_ctrl_; blue, R_Co_; green, R_Ni_; purple, R_SeW_.

The bacterial community composition analysis resulted in 861 unique ASV reads resulting from a total number of 9,982,381 raw reads. The bacterial diversity and evenness indices were similar among the samples collected during the stable operation (Figure 5a,b). After the onset of VFA accumulation in R_Ni_ and R_SeW_ at day 229 of operation, the first-order diversity (*^1^D*) of their bacterial communities began to increase, while the diversities in R_ctrl_ and R_Co_ remained relatively unchanged over the course of the experiment. The increase in first-order evenness (*^1^E*) of bacterial communities in R_Ni_ and R_SeW_ lagged behind their *^1^D*, increasing only after day 285. Correspondingly, NMDS and relative abundance plots indicate that the communities of all four reactors remained similar up until the beginning of VFA accumulation in R_Ni_ and R_SeW_, when their bacterial communities began to diverge from those of R_ctrl_ and R_Co_ (from day 257 in Figure 5c,d). Despite this shift, the bacterial communities of R_Ni_ and R_SeW_ remained similar to one another, as indicated by the continued proximity of their respective points on the NMDS plot (Figure 5c) and the results from cluster analysis of the samples (Table S4).

Before the initiation of TE depletion, the bacterial communities in all reactors were dominated by Firmicutes and Synergistetes phyla (48–51% and 35–39% of bacteria, respectively), while Spirochaetae and Bacteroidetes phyla constituted 5–7% and 4–5% of total bacteria, respectively (Figure 5d). Firmicutes were represented almost entirely by the genus *Lachnospiraceae NK3A20* (47–50% of all bacteria), which belongs to the Clostridia class. The Synergistetes were mostly dominated by the *Thermovirga* genus (24–30% of bacteria) and unidentified members of the Synergistaceae family (9–11% of bacteria). Running these sequences in the Basic Local Alignment Search Tool (BLAST, National Centre for Biotechnology Information, Bethesda, MD, USA) showed that they resembled members of the *Aminomonas* (90% identity), *Thermoanaerovibrio* (87% identity), and *Cloacibacillus* (91% identity) genera. Only two ASVs from the Spirochaetaceae family accounted for the vast majority (>99%) of Spirochaetae. Running these sequences in BLAST revealed that they were associated with the *Sphaerochaeta* genus (84% identity). Bacteroidetes was mostly dominated by members of the *Petrimonas* genus (50–59% of Bacteroidetes) and VadinBC27 wastewater-sludge group (21–25% of Bacteroidetes). 

The shifts in bacterial communities of R_Ni_ and R_SeW_ after VFA accumulation were primarily characterised by an increased diversity in the Synergistetes phylum. This manifested itself as a decreasing abundance of *Thermovirga* from 28% ± 1.2% to 4.7% ± 0.6% (*p* < 0.05, Student’s *t*-test) in R_Ni_ and from 29% ± 0.6% to 8.7% ± 0.6% (*p* < 0.05, *t*-test) in R_SeW_, and an increased abundance of *Aminobacterium*, *Pyramidobacter*, and *Synergistes*, reaching 2.0% ± 0.0%, 10% ± 1.5%, and 4.7% ± 0.6% in R_Ni_ and 7.7% ± 0.6%, 12% ± 0.6%, and 4.0% ± 1.0% of the respective bacterial sequence reads in R_SeW_. At the same time, the relative abundance of *Lachnoclostridium* increased from <1.0% in both reactors to 9.0% ± 1.0% and 4.3% ± 0.6% of bacteria in R_Ni_ and R_SeW_, respectively, while the abundance of members of the Spirochaetaceae family decreased from around 7.0% to <1.0% of bacteria. The relative abundances of *Lachnospiraceae NK3A20* decreased slightly, but not significantly during the experiment from 46% ± 1.0% to 37% ± 4.9% (*p* = 0.08, *t*-test) in R_Ni_ and from 46% ± 3.8% to 41% ± 4.9% (*p* = 0.24, *t*-test) in R_SeW_. Finally, *Petrimonas* was observed to have a decreasing trend in both reactors throughout the experiment from 3.0% ± 0.0% to <1.0% in R_Ni_, and from 2.7% ± 0.6% to <1.0% in R_SeW_.

### 3.4. Effects of TE Deficiency on Archaeal Community

**Figure 6:**
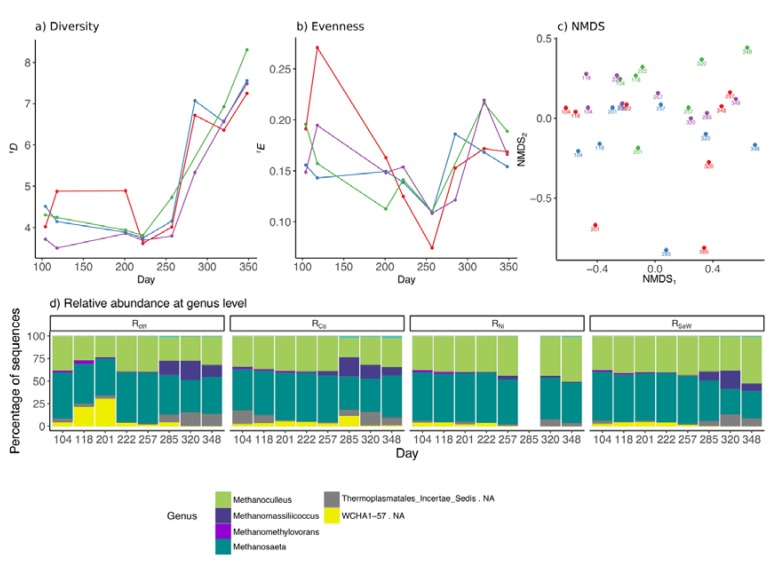
Results of the archaeal community analyses: (**a**) Hill diversity of order 1 (^1^D); (**b**) evenness of Hill order 1 (^1^E); (**c**) NMDS results; (**d**) relative abundances for each reactor separately at the genus level. Legend for (**a–c**), colours denote reactors: red, R_ctrl_; blue, R_Co_; green, R_Ni_; purple, R_SeW_. Data from reactor R_Ni_ on day 285 contained a large proportion of errors and were therefore removed from the analyses.

The archaeal community analysis resulted in a total of 623 unique ASV reads from an initial 7,829,605 raw sequence reads. Overall, no considerable differences were observed in the archaeal community compositions of R_Ni_ and R_SeW_ as compared to those of R_ctrl_ and R_Co_. The archaeal diversity (*^1^D*) and evenness (*^1^E*) remained relatively stable before TE depletion (Figure 6a,b), with the control reactor containing a more diverse and even community than the other reactors. After the initiation of the TE depletion, the *^1^D* of archaeal communities in all reactors increased, while the *^1^E* remained relatively constant until the end of the experiment. NMDS of the archaeal ASV reads did not reveal any discernible patterns throughout the experiment, and the relative abundances of most genera were comparable between the reactors (Figure 6c,d). *Methanosaeta* and *Methanoculleus* dominated in all reactors for the duration of the experiment (Figure 6d). During stable period, *Methanosaeta* represented 52% ± 2.8% of all archaeal sequence reads, followed by *Methanoculleus* at 38% ± 2.1% of archaea in all reactors, except R_ctrl_, where WCHA1-57 had a higher relative abundance (30% in R_ctrl_, as opposed to 3–6% in the other reactors; Figure 6d). After day 201, the relative abundance of this genus declined gradually, reaching the same levels as in the other reactors. A rapid increase of *Methanomassiliicoccus* genus and an unidentified genus of the Thermoplasmatales *incertae sedis* family was observed in R_ctrl_ and R_Co_ from day 285 and onwards (Figure 6d). A similar observation was made in R_SeW_, where these two groups reached 20% ± 1.2% and 33% ± 1.5%, respectively, on day 320. The sample from R_Ni_ on day 285 was excluded from the results due to a high likelihood of errors as evidenced by a high content (>50%) of sequences assigned to bacterial genera. The clustering algorithm highlighted that samples of the R_Ni_ community clustered together (cluster 10 in Table S5) between days 257 and 348, while R_SeW_ grouped together with the samples of R_ctrl_ and R_Co_ until just before the increase in butyrate concentration and formed its own separate cluster on day 348 (cluster 7 in Table S5).

## 4. Discussion

**Figure 7:**
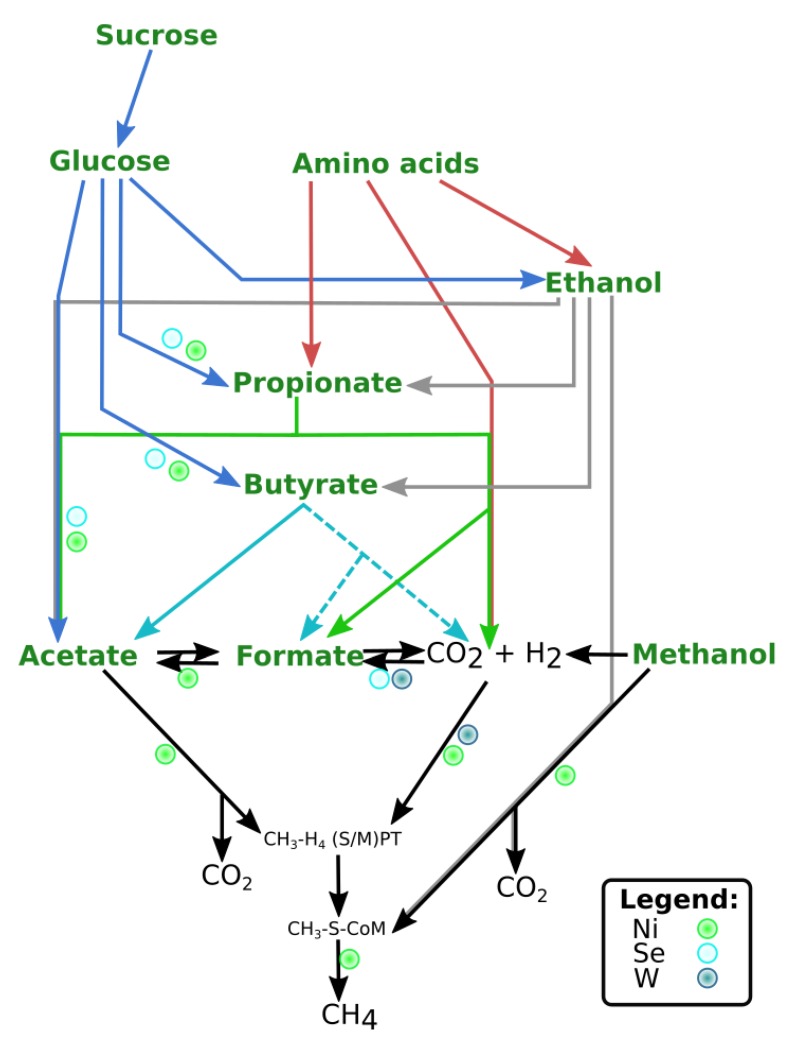
Schematic representation of the main metabolic pathways in the reactors in this study. The coloured dots represent which pathways may have been affected by lack of specific trace elements (TEs). Compounds marked with green bold text were present in the substrate fed to the reactors (based on [4,24,45]).

The results show that the process performances, microbial community compositions, and overall functions of the reactors in this study were similar during the experimental period before TE depletion. While there were some minor differences in gas production between the reactors, they were constant, suggesting that they likely occurred due to measuring uncertainties associated with each gas meter and methane sensor. Overall, the results from this period show that the majority of substrate was consumed between the daily feedings, as demonstrated by high VFA turnover (Figure 2 and Figure 3). This implies a kinetically balanced fermentation, acetogenesis, and methanogenesis. The stable operation regime was sustained for approximately 6 HRTs, showing that steady state conditions had been established before imposing the TE depletion to R_Co_, R_Ni_, and R_SeW_. During this time an average of 56% ± 4% of the theoretical methane production was achieved. Assuming TVS in these systems represents mainly microbial cells, it can be concluded that around 15% of ingoing COD was transformed to microbial biomass and related material, such as extracellular polymeric substances (EPS). The remaining 25–33% may have remained partially undegraded, or may have been lost through volatile compounds in the gas (e.g., VFA or H_2_), be transformed into some of the fermentation intermediates that were not monitored (e.g., lactate or alcohols), or left the reactor as dissolved CH_4_ and CO_2_ in the sludge. Based on the substrate composition and microbial community during the stable operation, we can deduce the most probable metabolic pathways prevailing in the reactors (Figure 7). The prevalent *Sphaerochaeta* and *Petrimonas* genera were likely responsible for fermentation of glucose and sucrose in the substrate to ethanol, acetate, formate, CO_2_, and hydrogen [39,40,41]. The amino acids, originating from the hydrolysed casein in the substrate, likely contributed to the proliferation of *Thermovirga*, which can ferment proteinaceous substrates and few organic acids, but not carbohydrates, fatty acids, or alcohols. In the case of casein fermentation, *Thermovirga* produces mainly ethanol, acetate, propionate, isovalerate, hydrogen, and CO_2_ [42]. The propionate and butyrate were likely oxidised by *Syntrophobacter sulfatireducens* and *Syntrophomonas wolfei* in syntrophic association with the methanogens as described in detail by Müller et al. [43], although these bacteria were detected with low relative abundances (<1% of bacterial ASV reads). Ethanol was most likely transformed to acetate and propionate, since these reactions are slightly more exergonic than the transformation to butyrate. The predominance of these reactions over the transformation to butyrate has been observed before for AD of sewage sludge [44].

The acetate was transformed to methane and CO_2_, presumably by the acetoclastic *Methanosaeta* [46], while formate, CO_2_, and hydrogen were likely transformed to methane by the hydrogenotrophic *Methanoculleus* [47]. The presence of methylotrophic *Methanomethylovorans* may be related to the supply of methanol in the substrate, which is likely converted to methane and CO_2_ [48,49]. While methanol may also be converted to acetate and butyrate, or CO_2_ and hydrogen, these pathways were likely much less active. The methanol concentration in the substrate was relatively low (around 20 mM), which would give a competitive advantage to methylotrophic methanogens, due to their higher affinity to methanol as compared to acetogenic bacteria [50]. From day 285 on, *Methanomassiliicoccus* increased in relative abundance, becoming the most abundant methylotrophic methanogen. This genus produces methane from H_2_ and methanol [51], suggesting that it may have taken over part of the function of *Methanoculleus* as the H_2_ consumer in the system (Figure 6). It should be noted that while relative abundances represent the general community dynamics in the reactor, they do not contain information on the metabolic activity of specific microorganisms. As a result, certain less abundant groups may have been more metabolically active than the groups discussed above. 

As Co is an essential component in many enzymes [7,8], the insignificant effect on process performance after its depletion indicates the possibility that sufficient amount of Co was entering the reactor with substrate components. An analysis of the Co content in different substrate components revealed that particularly casein contained Co (contributing around 540 ngCo L_substrate_^−1^; Table 1). Thus, the similar performance of R_ctrl_ and R_Co_ throughout the experiment indicated that Co content of the casein acted as a sufficient source of Co for the microbial activities. This corresponded to around 11 ng Co gCOD_fed_^−1^, which was apparently enough to maintain the metabolic activities of the fermentative, acetogenic, and methanogenic community of R_Co_. Casein also contained significant amounts of Ni, Se, and W (contributing around 26 µg Ni L_substrate_^−1^, 3 µg Se L_substrate_^−1^, and 230 ng W L_substrate_^−1^; Table 1). Nevertheless, the content of these TEs in casein proved to be either insufficient or unavailable for microbial activities, as removal of NiCl_2_ and Na_2_SeO_3_/Na_2_WO_4_ from the substrate lead to perturbation of the process in R_Ni_ and R_SeW_, respectively. 

In both R_Ni_ and R_SeW_, the TE depletion initially led to an increase in the values of the breakpoints (Figure 4b), implying that conversion of substrate to biogas and VFA turnover became kinetically restrained along with a decrease in TE concentration in the reactors. As the breakpoint time increased towards 24 h (Figure 4), propionate accumulation was observed in R_Ni_ and R_SeW_. The changes in the breakpoint parameter in response to TE depletion were observed approximately one week before propionate accumulation. This observation suggests that the kinetics of VFA conversion to biogas were already slowing down before this would manifest itself as VFA accumulation, which is commonly used as a sign of process disturbances [52]. Since no significant shifts in the microbial community compositions occurred in R_Ni_ and R_SeW_ before propionate accumulation, while the VFA and methane production kinetics were upset, the Ni and Se/W depletion apparently merely affected the activity of the community. It can therefore be presumed that the Ni and Se/W concentrations did not act as a selective factor for the microbial community compositions in R_Ni_ and R_SeW_. Nevertheless, while the effects on the abundance of dominant bacteria and archaea were marginal, the effects on the low-abundant syntrophic microorganisms (<1% of ASV reads) were substantial. Their low abundance, however, makes it difficult to make reliable conclusions on their dynamics.

To further pinpoint the specific metabolic pathways that were likely disturbed by Ni and Se/W depletion, the TEs required for the functioning of specific metabolic enzymes involved in substrate conversion in the reactors are shown in Figure 7. The gas kinetics were the first parameter to be observed to change, followed by propionate as the first monitored VFA that started to accumulate in both R_Ni_ and R_SeW_ as a response to TE depletion, suggesting that the methylmalonyl–CoA pathway (green arrows in Figure 7) was initially affected along with the declining TE concentrations [43]. Propionate oxidation requires low levels of formate or hydrogen [53], suggesting that product inhibition caused by high levels of these compounds may be a reason behind propionate accumulation. In fact, conversion of hydrogen via hydrogenotrophic methanogenesis and/or to formate requires Ni, Se, and W (Figure 7) [4,24,45]. 

Acetate started to accumulate when the propionate level was stabilised in both R_Ni_ and R_SeW_. This is very similar to the observations described by Takashima et al. [17], who explain that this may be due to the inhibition of acetoclastic methanogens by the increased propionate concentrations. Acetate reached a higher concentration in R_SeW_ than in R_Ni_, implying that either the acetate consumption was limited to a higher extent in R_SeW_ compared to that in R_Ni_, or acetate production was decreased in R_Ni_. It is not possible to state the exact reason behind the differences in acetate concentrations in R_SeW_ compared to those in R_Ni_ due multiple roles of Se/W and Ni in microbial metabolism. However, it may be argued that since Ni is vital for acetyl-CoA synthase/CO-dehydrogenase [54], as well as hydrogenases in general (present in all pathways going to acetate, propionate, and butyrate in Figure 7) [4,24,45], its depletion may have slowed down acetate production in R_Ni_, resulting in its comparatively lower concentrations compared to R_SeW_. 

The period after propionate accumulation was associated with substantial shifts in bacterial community composition in R_Ni_ and R_SeW_, which began to diverge from those in R_ctrl_ and R_Co_. However, the bacterial communities of R_Ni_ and R_SeW_ remained similar to one another throughout the experiment (Figure 5c and Table S4). The fact that the communities remained relatively similar in both R_Ni_ and R_SeW_ regardless of which TE was removed suggests that the major changes in bacterial community occurred as a response to VFA accumulation and not directly due to the TE depletion (e.g., due to VFA toxicity at low pH or product inhibition caused by VFA accumulation; [52]). An alternative possibility is that the depletion of the different TEs affected the same metabolic pathways and organisms in both reactors. More specifically, the explanation behind this might be that the initial increasing concentrations of H_2_ and formate due to TE depletion in both cases made the methylmalonyl–CoA pathway thermodynamically unfavourable, thus causing propionate accumulation [53]. Propionate eventually reached inhibitory levels for acetoclastic methanogens, causing acetate to accumulate [17]. The increasing accumulation of intermediates forced the microbial community to use other possible electron sinks in order to generate adenosine triphosphate (ATP), resulting in the accumulation of comparatively longer VFAs, such as, butyrate, as well as likely other compounds, such as e.g., lactate, ethanol, and butanol [55,56]. This contributed to the eventual pH drop, which after approaching each acid’s pKa, would cause a substantial portion of it to become undissociated and increase its inhibitory effect on the microbial community. The increased H_2_ concentrations in combination with the diverged electron flows to longer VFAs, may have caused an imbalance in the cells between NAD+ and NADH towards the former, possibly pushing the community to channel electrons from the assimilatory pathways to reduce their oxidised cofactors (i.e., NAD+ and oxidised ferredoxin to NADH and reduced ferredoxin, respectively) [57]. The consequence of maintaining energy metabolism at the expense of assimilatory pathways would be a decrease in biomass growth, leading to the washout of the most sensitive microorganisms and a corresponding increased relative abundance of the more resistant genera. In particular, *Lachnoclostridium,* a member of Clostridia, started replacing *Sphaerochaeta* of the Spirochaetae phylum during the period of acetate accumulation and later became one of the genera significantly associated with increasing VFA levels and process instabilities (samples from days 320 and 348). This result suggests that *Lachnoclostridium* may be more resistant to increased VFA and H_2_ concentrations than *Sphaerochaeta*. Most members of *Lachnoclostridium* are strict anaerobes, fermenting mono- and disaccharides mainly to acetate, while *Sphaerochaeta* tend to produce a broader range of compounds, such as ethanol, acetate, and formate [40,58]. 

Minor changes within the archaeal community were observed throughout the experiment, particularly a lower abundance of *Methanomassiliicoccus* in the Ni-depleted medium of R_Ni_, compared to the other reactors. This observation suggests a higher sensitivity of *Methanomassiliicoccus* to Ni depletion compared to that of the other methanogens. No significant response of this genus to Se/W depletion was observed despite the reported dependence of at least some of its members on these elements [51]. 

In conclusion, the observations in this study revealed that both Ni and Se/W depletion disturbed the VFA turnover, which according to information in the literature was most likely through disruption of H_2_ and/or formate utilisation. The microbial community composition, however, was initially not significantly affected, suggesting that their metabolic activities were merely progressively slowed down, as supported by the methane kinetics data. This result implies that reacting appropriately at the time of observed methane kinetics change, e.g., by increased TE supplementation, might allow for process recovery before VFAs begin to accumulate in the system. Due to the slower VFA turnover, propionic acid accumulated, followed by acetic acid, with the accumulation of both leading to a shift of carbon flows towards more reduced fermentation products, such as butyrate. The bacteria that were sensitive to this environment got washed out, leading to gradual shifts in their community with more resistant groups taking over.

## Figures and Tables

**Table 1:** Calculated concentrations of Co, Ni, Se, and W, as contributed by the different substrate components (ng/L), rounded to two significant digits.

Source	Co	Ni	Se	W
Glucose	1	63	75	0
Sucrose	6	75	1100	0
Casein	540	26,000	3000	230
Tap water	8	210	14	0
Vitamin solution	2	0	2	0
Trace element solution	20	13	4	2
Se/W solution	0	0	7	12
**Total**	**580**	**27,000**	**4200**	**250**

## Supplementary Materials

The following are available online at https://www.mdpi.com/2076-2607/8/4/598/s1, Table S1: Composition of the chemically defined substrate; Table S2: Number of sequence reads, obtained with primer set 515′F/805R, remaining for each sample at each step of the DADA2 pipeline; Table S3: Number of sequence reads, obtained with primer set 516F/915R, remaining for each sample at each step of the DADA2 pipeline; Table S4: Results of K-means clustering based on bacterial amplicon sequence variant (ASV) reads; Table S5: Results of K-means clustering based on archaeal ASV reads; Text S1: Next-generation amplicon sequence data processing.
