# Supplementary material for: Effect of Cobalt, Nickel, and Selenium/Tungsten Deficiency on Mesophilic Anaerobic Digestion of Chemically Defined Soluble Organic Compounds
Source: Microorganisms. 2020 Apr 20;8(4):598. doi: 10.3390/microorganisms8040598 (PMC7232481; doi:10.3390/microorganisms8040598)
Supplement: Supplementary file 1 [file microorganisms-08-00598-s001.pdf]

Effect of cobalt, nickel, and selenium/tungsten deficiency on mesophilic anaerobic digestion of chemically defined soluble organic compounds

L. Šafarič, S. Shakeri Yekta, B. H. Svensson, A. Schnürer, D. Bastviken, A. Björn

SUPLEMENTARY INFORMATION

Supplementary elements:

Text S1. Next generation amplicon sequence data processing
Table S1. Composition of the chemically defined substrate.
Table S2. Number of sequence reads, obtained with primer set 515'F/805R, remaining for each sample at each step of the DADA2 pipeline.
Table S3. Number of sequence reads, obtained with primer set 516F/915R, remaining for each sample at each step of the DADA2 pipeline.
Table S4. Results of K-means clustering based on bacterial ASV reads.
Table S5. Results of K-means clustering based on archaeal ASV reads.

Supplementary references.

Text S1: Next generation amplicon sequence data processing

Adapters were trimmed from the raw sequences with Cutadapt (Martin 2011). Sequences were filtered by removing those lacking adapters and those not having a length between 200 and 300 bp or between 250 and 500 bp for primer pairs 515'F/805R and 516F/915R, respectively. Sequences containing unspecified (i.e. N) bases were removed. The number of sequences after this step are presented as *Input* values in Tables S2 and S3.
The data was further processed with the DADA2 package in R (Callahan et al. 2016). Sequence read quality profiles were inspected, and the sequences filtered and trimmed with the *filterAndTrim* function. The truncation lengths were set to 220 and 170 bp for forward and reverse sequence reads with primers 515'F/805R, and to 245 and 155 bp for forward and reverse sequence reads of amplicons resulting from primers 516F/915R. The first 35 bases from reverse reads in amplicons from primers 515'F/805R were also trimmed due to low quality scores. The maximum expected errors were set to 2 for sequences obtained with both primer sets and all sequences were truncated when their quality score dropped to 11 or lower. The number of remaining sequences is presented as *Filtered* in Tables S2 and S3.
The model of error rates was learned from the data, followed by dereplication of the sequences. Sample inference was performed, resulting in the number of sequences, listed as *Denoised* in Tables S2 and S3, followed by merging of the paired ends. A sequence table was then constructed and the chimeras removed by selecting the "consensus" method. This resulted with the number of sequences presented as *Nochim.* in Tables S2 and S3. The resulting filtered data was analysed for taxonomy assignments with the Phyloseq package (McMurdie and Holmes 2013).

Table S1a. Composition of the chemically defined substrate. Compounds written in blue correspond to the buffer medium, green to carbon sources, yellow to vitamins, and red to trace elements. The stock solution was prepared by dissolving all the compounds in ultrapure water. It was then diluted to the final concentration by tap water in order to achieve the desired hydraulic retention time. The concentration of elements in the substrate originating from tap water are written in purple.

Substance	Chemical formula	Concentration in substrate (mM)
Monopotassium Phosphate	KH_2PO_4/H_2KO_4P	13
Sodium Bicarbonate	$NaHCO_3$	39
[a]Sodium Sulphate	Na_2SO_4	0.5
Ammonium Chloride	NH_4Cl	6.0
Sodium Chloride	$NaCl$	11
Magnesium Chloride Hexahydrate	$MgCl_2$ x $6H_2O$	1.3
Iron Chloride Tetrahydrate	$FeCl_2$ x $4H_2O$	0.2
Disodium Phosphate	Na_2HPO_4	14
Glucose	$C_6H_{12}O_6$	124
Sucrose	$C_{12}H_{22}O_{11}$	26
[a]Casein	[e]$C_{13}H_{25}O_7N_3S$	16
Methanol	CH_3OH	20
Ethanol	C_2H_6O	13
Acetic Acid	CH_3COOH	11
Propionic Acid	$C_3H_6O_2$	1.7
Butyric Acid	$C_4H_8O_2$	0.7
Formic Acid	CH_2O_2	1.4
Biotin (Vit. B8)	$C_{10}H_{16}N_2O_3S$	1.1E-07
[b]Vitamin B12	$C_{63}H_{88}CoN_{14}O_{14}P$	4.7E-08
P-aminobenzoic acid	$C_7H_7NO_2$	4.7E-07
Calcium D(+) Pantothenate (Vit. B5)	$C_{18}H_{32}CaN_2O_{10}$	1.3E-07
Thiamine Hydrochloride (Vit. B1)	$C_{12}H_{18}Cl_2N_4OS$	2.8E-07
Pyridoxine-HCl (Vit. B6)	$C_8H_{12}ClNO_3$	6.2E-07
Pyridoxamine-2HCl (Vit. B6)	$C_8H_{14}Cl_2N_2O_2$	1.3E-06
Nicotinamide	$C_6H_6N_2O$	1.1E-06
Nicotinic Acid (Niacin. Vit. B3)	$C_6H_5NO_2$	1.0E-06
Riboflavin (Vit. B2)	$C_{17}H_{20}N_4O_6$	1.7E-07
Folic Acid (Vit. B9)	$C_{19}H_{19}N_7O_6$	5.8E-08
Lipoic Acid	$C_8H_{14}O_2S_2$	3.1E-07
L-Ascorbic Acid (Vit. C)	$C_6H_8O_6$	7.3E-07
Boric Acid	H_3BO_3	8.3E-07
Manganese Sulphate Monohydrate	$MnSO_4$ x $1H_2O$	2.0E-07
[b]Cobalt Chloride Hexahydrate	$CoCl_2$ x $6H_2O$	2.7E-07
[c]Nickel Chloride Hexahydrate	$NiCl_2$ x $6H_2O$	1.3E-07
Cupric Chloride Dihydrate	$CuCl_2$ x $2H_2O$	2.3E-07
Zinc Chloride	$ZnCl_2$	3.8E-07
Ammonium Molybdate	$(NH_4)_6Mo_7O_{24}$ x $4H_2O$	5.2E-08
Aluminium Chloride Hydrate	$AlCl_3$ x H_2O	4.8E-07
[d]Sodium Selenite Pentahydrate	Na_2SeO_3 x $5H_2O$	7.3E-08
[d]Sodium Tungstate Dihydrate	Na_2WO_4 x $2H_2O$	7.8E-08
Chloride	Cl^-	1.2E-01 ± 8.7E-03
Sulphate	SO_4^{2-}	7.5E-02 ± 1.5E-02
Ammonium	NH_4^+	< 1.3E-03 ± < 2.6E-04
Nitrite	NO_2^-	< 2.6E-04 ± < 1.8E-04
Nitrate	NO_3^-	1.7E-02 ± 1.7E-03
Aluminium	Al	< 4.0 E-04 ± < 8.0E-05
Calcium	Ca	1.7E-01 ± 1.7E-02
Copper	Cu	< 2.8E-04 ± < 5.7E-05
Iron	Fe	< 1.3E-04 ± < 2.6E-05
Potassium	K	1.7E-02 ± 1.7E-03
Magnesium	Mg	3.7E-02 ± 3.7E-03
Manganese	Mn	< 3.3E-05 ± < 3.3E-06
Sodium	Na	1.3E-01 ± 1.3E-02

[a] Sodium sulphate and hydrolysed casein served as precursors for sulphide and sulphur sources (Muyzer and Stams 2008).
[b] Vitamin B12 and cobalt chloride were removed from the substrate of R2 during the trace element depletion experiment.
[c] Nickel chloride was removed from the substrate of R3 during the trace element depletion experiment.
[d] Sodium selenite and sodium tungstate were removed from the substrate of R4 during the trace element depletion experiment.
[e] The chemical formula of hydrolysed casein was assumed to be the generic elementary formula for amino acids in proteins, since the exact composition was not known (Speda et al. 2016).

Table S1b. Content of Co, Ni, Se, and W in the main carbon sources in ng/g. N.D. – not detected

Substance	Co	Ni	Se	W
Glucose	1	3	3	N.D.
Sucrose	>1	8	125	N.D.
Casein	43	2114	239	19

Table S2. Number of sequence reads, obtained with primer set 515'F/805R, remaining for each sample at each step of the DADA2 pipeline. *Input* – number of raw sequences; *Filtered* – number of sequences after filtering; *Denoised* – number of sequences after sample inference; *Merged* – number of sequences after merging the forward- and reverse reads; *Tabled* – reads after constructing the sequence table; *Nochim.* – number of reads remaining after removal of chimeras.

Sample ("Reactor" + "day")	Input	Filtered	DenoisedF	DenoisedR	Merged	Tabled	Nonchim
**R_{ctrl} 104**	158927	13818	13818	13818	11654	11654	7366
**R_{ctrl} 104**	154613	11999	11999	11999	10072	10072	6444
**R_{ctrl} 104**	178433	13470	13470	13470	11368	11368	7200
**R_{Co} 104**	113688	8665	8665	8665	7411	7411	5430
**R_{Co} 104**	137705	11683	11683	11683	9804	9804	6978
**R_{Co} 104**	154627	17286	17286	17286	14729	14729	9805
**R_{Ni} 104**	107481	7526	7526	7526	6046	6046	3739
**R_{Ni} 104**	115466	7805	7805	7805	6318	6318	4137
**R_{Ni} 104**	121365	11619	11619	11619	9681	9681	6143
**R_{SeW} 104**	123756	11800	11800	11800	10169	10169	6523
**R_{SeW} 104**	189533	22300	22300	22300	19226	19226	12135
**R_{SeW} 104**	182292	20030	20030	20030	17220	17220	11170
**R_{ctrl} 118**	169611	18997	18997	18997	16072	16072	9823
**R_{ctrl} 118**	135483	14787	14787	14787	12440	12440	7622
**R_{ctrl} 118**	97679	9749	9749	9749	8182	8182	5063
**R_{Co} 118**	74659	8405	8405	8405	7024	7024	4707
**R_{Co} 118**	88194	10534	10534	10534	8939	8939	5777
**R_{Co} 118**	182893	18326	18326	18326	15523	15523	9760
**R_{Ni} 118**	238508	27064	27064	27064	22598	22598	13387
**R_{Ni} 118**	209675	23488	23488	23488	19650	19650	11749
**R_{Ni} 118**	82356	8553	8553	8553	7133	7133	4196
**R_{SeW} 118**	126356	12070	12070	12070	10181	10181	6626
**R_{SeW} 118**	133268	12598	12598	12598	10758	10758	6904
**R_{SeW} 118**	178808	16961	16961	16961	14494	14494	9080
**R_{ctrl} 201**	169045	17526	17526	17526	14568	14568	9244
**R_{ctrl} 201**	134769	15003	15003	15003	12785	12785	8223
**R_{ctrl} 201**	79745	5539	5539	5539	4757	4757	3413
**R_{Co} 201**	134292	14373	14373	14373	12194	12194	7759
**R_{Co} 201**	119238	6050	6050	6050	4889	4889	3492
**R_{Co} 201**	116409	10327	10327	10327	8819	8819	5884
**R_{Ni} 201**	132992	14956	14956	14956	12450	12450	8038
**R_{Ni} 201**	151874	15755	15755	15755	13414	13414	8591
**R_{Ni} 201**	136832	14766	14766	14766	12394	12394	7913

**R_{SeW} 201**	88921	9403	9403	9403	7879	7879	5032
**R_{SeW} 201**	106463	10907	10907	10907	9682	9682	7037
**R_{SeW} 201**	61586	6676	6676	6676	5584	5584	3935
**R_{ctrl} 222**	89579	10649	10649	10649	8997	8997	5754
**R_{ctrl} 222**	95562	9467	9467	9467	7989	7989	5332
**R_{ctrl} 222**	132211	14374	14374	14374	11998	11998	7588
**Rco 222**	103469	11576	11576	11576	9687	9687	6129
**Rco 222**	122520	12248	12248	12248	10206	10206	6384
**Rco 222**	75806	7122	7122	7122	6055	6055	4239
**R_{Ni} 222**	91907	8601	8601	8601	7361	7361	5035
**R_{Ni} 222**	122603	11022	11022	11022	9243	9243	5981
**R_{Ni} 222**	87619	9005	9005	9005	7639	7639	4976
**R_{SeW} 222**	95486	10507	10507	10507	8972	8972	5909
**R_{SeW} 222**	81636	5376	5376	5376	4566	4566	3200
**R_{SeW} 222**	86359	9017	9017	9017	7602	7602	5167
**R_{ctrl} 257**	54226	5084	5084	5084	4245	4245	3278
**R_{ctrl} 257**	31708	1614	1614	1614	1418	1418	1162
**R_{ctrl} 257**	63025	7160	7160	7160	6038	6038	4490
**R_{Co} 257**	109761	11031	11031	11031	9478	9478	6455
**R_{Co} 257**	56275	5923	5923	5923	5067	5067	3605
**R_{Co} 257**	78241	8101	8101	8101	6803	6803	4759
**R_{Ni} 257**	89799	8116	8116	8116	6720	6720	4953
**R_{Ni} 257**	81213	8555	8555	8555	7132	7132	4974
**R_{Ni} 257**	66245	7449	7449	7449	6179	6179	4329
**R_{SeW} 257**	111650	10742	10742	10742	8887	8887	6086
**R_{SeW} 257**	83454	9150	9150	9150	7535	7535	5137
**R_{SeW} 257**	79176	8557	8557	8557	7117	7117	5155
**R_{ctrl} 285**	85251	8440	8440	8440	7003	7003	5009
**R_{ctrl} 285**	93348	8488	8488	8488	7162	7162	4996
**R_{ctrl} 285**	86481	7539	7539	7539	6396	6396	4621
**R_{Co} 285**	90529	7887	7887	7887	6610	6610	4672
**R_{Co} 285**	81603	8051	8051	8051	6832	6832	4956
**R_{Co} 285**	119077	14896	14896	14896	12526	12526	8176
**R_{Ni} 285**	76794	5818	5818	5818	5064	5064	4410
**R_{Ni} 285**	126401	14786	14786	14786	13098	13098	10930
**R_{Ni} 285**	108018	11299	11299	11299	9953	9953	8470
**R_{SeW} 285**	105554	10803	10803	10803	9198	9198	6400
**R_{SeW} 285**	106180	11076	11076	11076	9054	9054	6358
**R_{SeW} 285**	76877	8911	8911	8911	7340	7340	5396
**R_{ctrl} 320**	67138	8513	8513	8513	7087	7087	4723
**R_{ctrl} 320**	50341	6232	6232	6232	5231	5231	3728
**R_{ctrl} 320**	43874	5053	5053	5053	4276	4276	3288
**R_{Co} 320**	87816	11036	11036	11036	10112	10112	8038
**R_{Co} 320**	43684	5729	5729	5729	4987	4987	3488
**R_{Co} 320**	61740	6966	6966	6966	5961	5961	4515
**R_{Ni} 320**	85300	10444	10444	10444	8649	8649	6254
**R_{Ni} 320**	112057	13766	13766	13766	11256	11256	8006
**R_{Ni} 320**	91577	10328	10328	10328	8447	8447	5968
**R_{SeW} 320**	141143	16017	16017	16017	13410	13410	9518
**R_{SeW} 320**	91364	9676	9676	9676	8198	8198	6005
**R_{SeW} 320**	83999	8899	8899	8899	7463	7463	5743
**R_{ctrl} 348**	82892	9511	9511	9511	8302	8302	6049
**R_{ctrl} 320**	119971	12734	12734	12734	10934	10934	7850
**R_{ctrl} 348**	94283	5986	5986	5986	5190	5190	3957
**R_{Co} 348**	93210	9569	9569	9569	8245	8245	5524
**R_{Co} 348**	50717	4616	4616	4616	3959	3959	2748
**R_{Co} 348**	70541	6150	6150	6150	5459	5459	3813
**R_{Ni} 348**	79887	9055	9055	9055	6915	6915	4963
**R_{Ni} 348**	69589	4450	4450	4450	3446	3446	2819

**R_{Ni} 348**	68810	7437	7437	7437	5688	5688	4201
**R_{SeW} 348**	50090	5309	5309	5309	4320	4320	3265
**R_{SeW} 348**	54742	5163	5163	5163	4230	4230	3349
**R_{SeW} 348**	54431	5839	5839	5839	4770	4770	3787

Table S3. Number of sequence reads, obtained with primer set 516F/915R, remaining for each sample at each step of the DADA2 pipeline. *Input* – number of raw sequences; *Filtered* – number of sequences after filtering; *Denoised* – number of sequences after sample inference; *Merged* – number of sequences after merging the forward- and reverse reads; *Tabled* – reads after constructing the sequence table; *Nochim.* – number of reads remaining after removal of chimeras.

Sample ("Reactor" + "day")	input	filtered	denoisedF	denoisedR	merged	tabled	nonchim
**R_{ctrl} 104**	51319	15095	15095	14029	14029	11037	51319
**R_{ctrl} 104**	54055	14861	14861	13861	13861	10397	54055
**R_{ctrl} 104**	86558	18259	18259	17033	17033	12701	86558
**R_{Co} 104**	50803	14112	14112	12719	12719	9853	50803
**R_{Co} 104**	59496	16989	16989	14928	14928	11266	59496
**R_{Co} 104**	69873	22102	22102	19852	19852	15285	69873
**R_{Ni} 104**	53211	10975	10975	9969	9969	7854	53211
**R_{Ni} 104**	66846	19726	19726	17983	17983	13052	66846
**R_{Ni} 104**	53562	15383	15383	14101	14101	10333	53562
**R_{SeW} 104**	60055	17679	17679	15905	15905	11680	60055
**R_{SeW} 104**	99669	31592	31592	28224	28224	21101	99669
**R_{SeW} 104**	73988	23508	23508	20903	20903	15057	73988
**R_{ctrl} 118**	114075	30000	30000	15156	15156	13040	114075
**R_{ctrl} 118**	69332	17187	17187	8575	8575	7555	69332
**R_{ctrl} 118**	96799	22554	22554	10900	10900	9271	96799
**R_{Co} 118**	104624	33178	33178	30380	30380	23180	104624
**R_{Co} 118**	85404	28157	28157	25880	25880	18631	85404
**R_{Co} 118**	81191	21139	21139	19127	19127	14645	81191
**R_{Ni} 118**	131757	40310	40310	37148	37148	30331	131757
**R_{Ni} 118**	101315	32910	32910	29978	29978	24658	101315
**R_{Ni} 118**	89401	27488	27488	25185	25185	19529	89401
**R_{SeW} 118**	57100	17094	17094	15426	15426	11230	57100
**R_{SeW} 118**	36408	8669	8669	7865	7865	6461	36408
**R_{SeW} 118**	55494	16321	16321	14920	14920	12519	55494
**R_{ctrl} 201**	54002	15069	15069	5912	5912	4606	54002
**R_{ctrl} 201**	54640	13858	13858	5159	5159	3541	54640
**R_{ctrl} 201**	44781	9226	9226	3885	3885	2911	44781
**R_{Co} 201**	88728	28665	28665	25491	25491	20027	88728
**R_{Co} 201**	60443	13749	13749	12202	12202	9162	60443
**R_{Co} 201**	76197	21517	21517	18976	18976	15453	76197
**R_{Ni} 201**	114611	35157	35157	30709	30709	20892	114611
**R_{Ni} 201**	46322	14143	14143	12093	12093	8446	46322
**R_{Ni} 201**	84192	25956	25956	22858	22858	15181	84192
**R_{SeW} 201**	83642	26016	26016	23143	23143	17726	83642
**R_{SeW} 201**	53235	14019	14019	12442	12442	9738	53235
**R_{SeW} 201**	119191	37174	37174	33735	33735	26552	119191
**R_{ctrl} 222**	60308	19081	19081	17105	17105	13134	60308
**R_{ctrl} 222**	65953	16594	16594	14830	14830	11182	65953
**R_{ctrl} 222**	71375	21226	21226	19112	19112	15445	71375
**Rco 222**	101556	31871	31871	28923	28923	23079	101556
**Rco 222**	75429	22412	22412	20500	20500	15086	75429
**Rco 222**	50977	14670	14670	13347	13347	10409	50977
**R_{Ni} 222**	34168	7739	7739	7117	7117	5580	34168
**R_{Ni} 222**	93585	26524	26524	25047	25047	19419	93585

**R$_{Ni}$ 222**	83891	24799	24799	23112	23112	17425	83891
**R$_{SeW}$ 222**	71279	22048	22048	19976	19976	17721	71279
**R$_{SeW}$ 222**	39891	8198	8198	7353	7353	6471	39891
**R$_{SeW}$ 222**	48401	15412	15412	13948	13948	12001	48401
**R$_{ctrl}$ 257**	36773	10055	10055	8968	8968	6942	36773
**R$_{ctrl}$ 257**	38012	9278	9278	8204	8204	6520	38012
**R$_{ctrl}$ 257**	124711	38335	38335	35248	35248	27644	124711
**R$_{Co}$ 257**	129882	39464	39464	36429	36429	27708	129882
**R$_{Co}$ 257**	86414	26324	26324	24025	24025	18291	86414
**R$_{Co}$ 257**	94342	28576	28576	26031	26031	18407	94342
**R$_{Ni}$ 257**	89527	21791	21791	19827	19827	13909	89527
**R$_{Ni}$ 257**	95057	29041	29041	22838	22838	15621	95057
**R$_{Ni}$ 257**	82077	25923	25923	24249	24249	15671	82077
**R$_{SeW}$ 257**	121068	29998	29998	27306	27306	20276	121068
**R$_{SeW}$ 257**	152328	44947	44947	41918	41918	31739	152328
**R$_{SeW}$ 257**	126433	39458	39458	36929	36929	28473	126433
**R$_{ctrl}$ 285**	79931	23937	23937	9936	9936	6470	79931
**R$_{ctrl}$ 285**	60827	17556	17556	7101	7101	4599	60827
**R$_{ctrl}$ 285**	28315	6467	6467	2590	2590	1753	28315
**R$_{Co}$ 285**	77957	22326	22326	9625	9625	5963	77957
**R$_{Co}$ 285**	80737	21695	21695	9497	9497	5700	80737
**R$_{Co}$ 285**	55967	19292	19292	7608	7608	4997	55967
**R$_{Ni}$ 285**	67782	16809	16809	4850	4850	4726	67782
**R$_{Ni}$ 285**	85067	29634	29634	7463	7463	7212	85067
**R$_{Ni}$ 285**	73402	22561	22561	5773	5773	5521	73402
**R$_{SeW}$ 285**	46977	13956	13956	12787	12787	9286	46977
**R$_{SeW}$ 285**	122456	37296	37296	34515	34515	24534	122456
**R$_{SeW}$ 285**	172454	55838	55838	51754	51754	36655	172454
**R$_{ctrl}$ 320**	81102	25478	25478	21123	21123	16401	81102
**R$_{ctrl}$ 320**	72143	22678	22678	17915	17915	13808	72143
**R$_{ctrl}$ 320**	84987	23155	23155	19121	19121	14193	84987
**R$_{Co}$ 320**	145412	46118	46118	40825	40825	29749	145412
**R$_{Co}$ 320**	105647	34275	34275	29570	29570	22424	105647
**R$_{Co}$ 320**	68828	18401	18401	15810	15810	12001	68828
**R$_{Ni}$ 320**	29762	9270	9270	8668	8668	5875	29762
**R$_{Ni}$ 320**	86753	28900	28900	27380	27380	18011	86753
**R$_{Ni}$ 320**	150602	46725	46725	44218	44218	27984	150602
**R$_{SeW}$ 320**	82843	24074	24074	20885	20885	15410	82843
**R$_{SeW}$ 320**	43011	10285	10285	9146	9146	6688	43011
**R$_{SeW}$ 320**	102233	29151	29151	26277	26277	20364	102233
**R$_{ctrl}$ 348**	128692	39292	39292	35075	35075	26785	128692
**R$_{ctrl}$ 320**	96004	29890	29890	26577	26577	18808	96004
**R$_{ctrl}$ 348**	100206	20508	20508	17777	17777	11968	100206
**R$_{Co}$ 348**	125767	39955	39955	34232	34232	21357	125767
**R$_{Co}$ 348**	113137	31227	31227	26407	26407	16807	113137
**R$_{Co}$ 348**	77401	22314	22314	18505	18505	11422	77401
**R$_{Ni}$ 348**	67377	20838	20838	19435	19435	11414	67377
**R$_{Ni}$ 348**	70359	19351	19351	18159	18159	10608	70359
**R$_{Ni}$ 348**	100860	31510	31510	29622	29622	17622	100860
**R$_{SeW}$ 348**	117601	36133	36133	32994	32994	21991	117601
**R$_{SeW}$ 348**	65992	16843	16843	15254	15254	10138	65992
**R$_{SeW}$ 348**	105258	32586	32586	25450	25450	16896	105258

Table S4. Results of K-means clustering based on bacterial ASV reads.

Cluster	Samples ["Reactor" + "day"]	Significant genera	Indicator values	p
1	R_{Ni} 104	*Intestinibacter*	1	0.041
		Chishuiella	1	0.036
		Unknown member of LD1-PB3	1	0.031
		Solibacillus	1	0.008
2	R_{ctrl}320, R_{ctrl} 348, R_{Co} 320, R_{Co} 348	/	/	/
3	R_{ctrl} 201, R_{ctrl} 222, R_{Co} 201, R_{Co} 222, R_{Ni} 201, R_{Ni} 222, R_{SeW} 201, R_{SeW} 222	Unknown member of Elusimicrobia	0.57	0.002
4	R_{ctrl} 257, R_{ctrl} 258, R_{Co} 257, R_{Co} 258	/	/	/
5	R_{Ni} 320, R_{Ni} 348, R_{SeW} 320, R_{SeW} 348	*Aminobacterium*	0.85	0.001
		Synergistes	0.81	0.001
		Bacteroides	0.79	0.002
		Corynebacterium	0.67	0.049
		Comamonas	0.61	0.034
		Pyramidobacter	0.59	0.003
		Unknown member of Lachnospiraceae	0.47	0.019
		Lachnoclostridium	0.45	0.026
6	R_{ctrl} 104, R_{Co} 104	*Intestinimonas*	0.71	0.015
		Lactobacillus	0.63	0.013
		Unknown member of Sphingobacteriales	0.63	0.014
		Syntrophorhabdus	0.61	0.005
		Unknown member of Bacteriovoracaceae	0.47	0.027
		Unknown member of W5	0.39	0.002
		vadinBC27_wastewater-sludge_group	0.26	0.035
		Unknown member of Spirochaetaceae	0.24	0.009
7	R_{Ni} 257, R_{Ni} 285, R_{SeW} 257, R_{SeW} 285	Unknown member of Enterobacteriaceae	0.79	0.002
		Escherichia/*Shighella*	0.69	0.039
		Unknown member of Family_XIII	0.61	0.011
8	R_{ctrl} 118, R_{Co} 118, R_{Ni} 118, R_{SeW} 104, R_{SeW} 118	/	/	/

Table S5. Results of K-means clustering based on archaeal ASV reads.

Cluster	Samples ["Reactor" + "day"]	Significant genera	Indicator values	p
1	R_{Co} 104, R_{Co} 118	Unidentified members of Terrestrial_Miscellaneous_Gp(TMEG)	0.73	0.001
2	R_{ctrl} 285, R_{Co} 285	/	/	/
3	R_{Co} 201, R_{Ni} 104, R_{Ni} 118, R_{SeW} 118, R_{SeW} 201	/	/	/
4	R_{ctrl} 320, R_{ctrl} 348, R_{Co} 320, R_{SeW} 320	/	/	/
5	R_{ctrl} 222, R_{ctrl} 257, R_{Co} 222, R_{Ni} 222, R_{SeW} 222, R_{SeW} 257	/	/	/
6	R_{ctrl} 104, R_{Co} 257, R_{Ni} 201, R_{SeW} 104	/	/	/
7	R_{SeW} 348	Unidentified members of Euryarchaeota	1	0.027
		Unidentified members of Archaea	0.79	0.047
8	R_{ctrl} 118, R_{ctrl} 201	Unidentified members of WCHA1-57	0.33	0.035
9	R_{Co} 3480 R_{SeW} 285	/	/	/
10	R_{Ni} 257, R_{Ni} 320, R_{Ni} 348	/	/	/

REFERENCES

Callahan, Benjamin J, Paul J McMurdie, Michael J Rosen, Andrew W Han, Amy Jo A Johnson, and Susan P Holmes. 2016. “DADA2: High Resolution Sample Inference from Illumina Amplicon Data.” *Nature Methods* 13 (7): 581–83. https://doi.org/10.1038/nmeth.3869.

Martin, Marcel. 2011. “Cutadapt Removes Adapter Sequences from High-Throughput Sequencing Reads.” *EMBnet.Journal* 17 (1): 10. https://doi.org/10.14806/ej.17.1.200.

McMurdie, Paul J., and Susan Holmes. 2013. “Phyloseq: An R Package for Reproducible Interactive Analysis and Graphics of Microbiome Census Data.” *PLoS ONE* 8 (4). https://doi.org/10.1371/journal.pone.0061217.

Muyzer, Gerard, and Alfons J M Stams. 2008. “The Ecology and Biotechnology of Sulphate-Reducing Bacteria.” *Nature Reviews. Microbiology* 6 (6): 441–54. https://doi.org/10.1038/nrmicro1892.

Speda, Jutta, Mikaela A. Johansson, Bengt-Harald Jonsson, and Martin Karlsson. 2016. “Applying Theories of Microbial Metabolism for Induction of Targeted Enzyme Activity in a Methanogenic Microbial Community at a Metabolic Steady State.” *Applied Microbiology and Biotechnology*. https://doi.org/10.1007/s00253-016-7547-z.
